# Prevalent distribution and survival outcome of HPV infection in patients with early-stage cervical cancer in Hangzhou, China

**DOI:** 10.1186/s12879-022-07888-0

**Published:** 2022-12-15

**Authors:** Xiaoxian Xu, Tao Feng, Dan Li, Hanmei Lou, Huiyin Lan

**Affiliations:** 1 The Cancer Hospital of the University of Chinese Academy of Sciences (Zhejiang Cancer Hospital), Hangzhou, 310022 Zhejiang China; 2 Institute of Basic Medicine and Cancer (IBMC), Chinese Academy of Sciences, Hangzhou, 310022 Zhejiang China; 3 Zhejiang Chinese Medical University, Hangzhou, China; 4 Department of Radiation Oncology, The Second Affiliated Hospital of Xi’an Jiaotong University, Xi’an, Shanxi China; 5 Zhejiang Key Laboratory of Radiation Oncology, Hangzhou, 310022 Zhejiang China

**Keywords:** Genotype, High‐risk human papillomavirus (HR‐HPV), Cervical cancer, Overall survival (OS), Prevalence

## Abstract

**Objectives:**

To describe the prevalent distribution of human papilloma virus (HPV) infection in patients with early-stage cervical squamous cell carcinoma (CSCC). To provide data on high-risk HPV (HR-HPV) infection and other clinicopathological factors for their correlations with the survival of CSCC patients.

**Methods:**

A total of 1425 patients with FIGO stages IA to IIA CSCC who underwent radical surgery between September 2008 and December 2012 were enrolled in the study. The prevalent distribution of HPV infection with different patient characteristics and survivals were analyzed with or without propensity score matching (PSM).

**Results:**

The overall infection rate of HPV was 84.3%, including 13 carcinogenic HR-HPV genotypes and 8 low-risk HPV genotypes with infection rates of 82.6% and 5.8%, respectively. The distribution of HPV infection were proportional in patients with either different age groups or different FIGO stages. HPV16 was the dominant subtype with an infection rate of 65.1%, followed by the other top four subtypesHPV58 (8.7%), 18 (7.7%) and 52 (4.5%). χ^2^ analysis revealed that increased preoperative serum squamous cell carcinoma antigen levels and lymphovascular space invasion (LVSI) were statistically associated with HPV status. However, regression analyses indicated that only deep stromal invasion, LVSI and lymph node metastasis were independent prognostic factors on 5-year overall survival (OS), but not HR-HPV infection status even in the second exploratory analysis (*P* = 0.939) based on the PSM applied to reduce selection bias.

**Conclusions:**

This study provided baseline data on the prevalence characteristics of HPV infections in patients with early-stage CSCC, and HR-HPV infection was not a prognosticator of 5-year OS, other than FIGO stage, LVSI and lymph node metastasis.

## Introduction

Cervical cancer ranks as the fourth leading cause of both cancer incidence and mortality in females worldwide [1]. In China, cervical cancer contributed an estimated of 109,741 (18.17%) cases and 59,060 (17.28%) deaths in women according to a secondary analysis of the GLOBOCAN 2020 data [2]. Cervical squamous cell carcinoma (CSCC) is the most common histological type accounting for more than 70% of all cervical cancer cases in USA [3] and for more than 90% in China [4]. Epidemiological studies have well shown that persistent HPV infection is the main causative and necessary factor for the development of more than 91% of cervical cancer cases [5]. Based on their pathogenic potential, HPVs were generally classified into two subgroups: high-risk of carcinogenic HPV types (HR-HPV) and low-risk of carcinogenic HPV types (LR-HPV) [6]. Among the HR-HPV types, they were further divided into two species based on their genomic nucleotide similarity: alpha-7 (HPV18, 39, 45, 59, 68, and 70) and alpha-9 (HPV16, 31, 33, 35, 52, 58, and 67), accounting for over 80% of whole cervical cancer cases [7]. Usually it is well-recognized that HPV16 and 18are the most two common prevalent genotypes worldwide, which cause 70% of all cervical cancers, however, the prevalent distribution of the other HR-HPV genotypes varies in different countries by geographical regions. To specific, HPV45, 31, and 33 are preferentially prevalent in western countries, whereas HPV58 and 52 are more prevalent in Asian populations including Chinese [8]. To date, there are three efficacious HPV vaccines are available in the global market, including the bivalent vaccine (HPV16/18), the quadrivalent vaccine (HPV16/18/6/11) and the nonavalent vaccine (HPV16/18/6/11/31/33/45/52/58). Therefore, understanding the type-specific HPV distribution in certain geographic-specific regions is of great significance for correct selecting prophylactic vaccines and cervical cancer prevention.

For those patients with early-stage (FIGO IA-IIA) CSCC, surgical intervention is the preferred treatment modality [9]. However, the prognostic association of HR-HPV infection status with the survival of early-stage CSCC patients undergoing primary operation remains controversial. For example, Huang et al. reported that the presence of HPV16 and its related species alpha-9 predicted an improved survival [10]; Lai et al. found that HPV18 positivity predicted a worse recurrence-free survival (RFS) and OS, whereas the total HPV status and HPV pattern (single or multiple type) were unrelated to survival [11]; However, Zampronha Rde et al. showed thatHPV16 and 18 did not affect the prognosis of patients with stage I cervical [12]. Therefore, more clinical studies is needed to clarify the prognostic value of HPV infection on cervical cancers.

In this present study, we evaluated the prevalent distribution of HPV and analyzed the prognostic value of HR-HPV for 5-year survival in 1425 patients with early-stage CSCC who underwent radical surgery in Hangzhou.

## Methods

### Study population

**Fig. 1:**
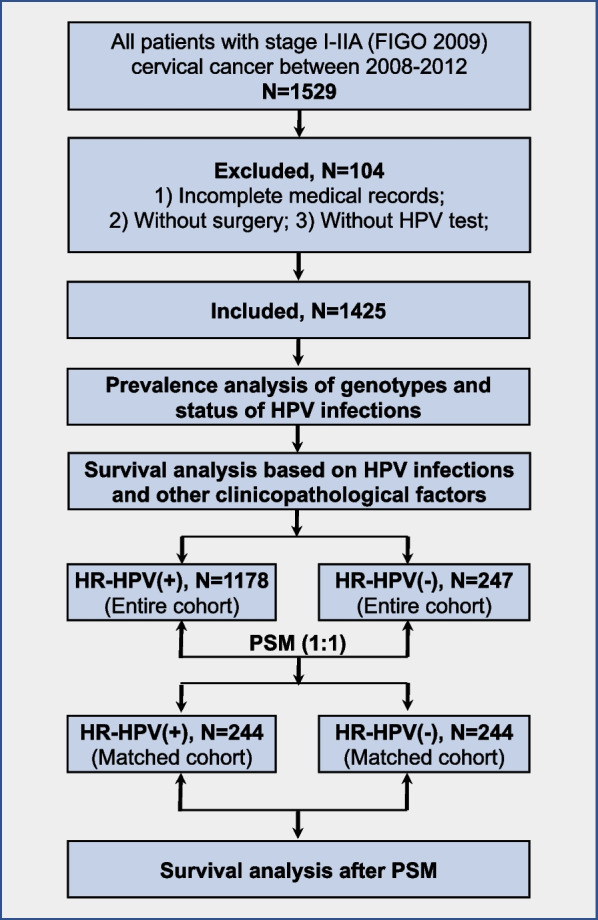
Study flowchart. *FIGO* International Federation of Gynecology and Obstetrics, *HR-HPV* high risk human papillomavirus, *PSM* propensity score matching, *SCC* squamous cell carcinoma

All consecutive patients diagnosed with FIGO (2009) stage I-IIA and histologically confirmed CSCC who underwent primary radical hysterectomies surgery (either conventional open surgery including type B, type C1, and type C2, or minimally invasive laparoscopic surgery) from September 2008 to December 2012 at Cancer Hospital of The University of Chinese Academy of Sciences, Hangzhou, China, were selected for this study. The exclusion criteria were as follows: (1) Patients without complete medical records; (2) Patients did not undergo primary surgery; (3) Patients without performed HPV genotyping test. Details of procedure for inclusion and exclusion resulting in the final study group are described in Fig. 1.

### Data collection

The demographic variables and clinicopathological parameters were extracted from medical records in the electronic database of the Cancer Hospital of the University of Chinese Academy of Sciences, including age at surgery, FIGO stage, histological report, differentiation grade and squamous cell carcinoma antigen (SCC-Ag) levels. Informed written consent was obtained from all patients. The Medical Ethics Committee of Cancer Hospital of the University of Chinese Academy of Sciences approved the study.

Follow-up data were obtained through correspondence and/or outpatient department visits. Overall survival (OS) was measured from the time between the date of diagnosis and the date of death or the last follow-up.

### Cytological sample collection and HPV genotyping

Samples of exfoliated cervical cells were collected using a disposable cytology brush (Bluesail Medical Co., Ltd., Ningbo, China) and dislodged into PreservCyt collection solution (Hologic, Inc., MA, USA) and stored at 4 °C until processed for DNA extraction.

13 HR-HPV genotypes (HPV16, 18, 31, 33, 35, 39, 45, 51, 52, 56, 58, 59 and 68) and 8 LR-HPV genotypes (HPV6, 11, 44, 51, 53, 66, 68 and CPB304) were genotyped by HybriMax using a HPV GenoArray Test Kit (HybriBio Ltd., Chaozhou, China) according to the manufacturer’s instructions [13]. The PCR procedure has been described in our previous study [14]. Briefly, PCR was performed in a 25 μL reaction mixture containing 5 μL DNA template, 0.75 μL DNA Taq polymerase and 19.25 μL PCR-mix solution containing MY09/11 primer system. The PCR program was: denaturation at 95 °C for 9 min, followed by 40 cycles at 95 °C for 20 s, 55 °C for 30 s, and 72 °C for 30 s, and finally extension at 72 °C for 5 min. A positive control and a negative control were included in each PCR analysis process to control for possible contamination and accuracy.

### Postoperative adjuvant therapy

Patients with postoperative pathological risk factors, such as lymphatic metastases, parametrial and surgical margin involvement, or comply with Sedlis criteria were advised to receive adjuvant radiotherapy (RT) or concurrent chemoradiotherapy (CCRT). Pelvic external beam RT was performed with a total of 45–50 Gy [15].

### Patient and public involvement

Neither patients nor the public were involved in setting the research questions or the design of the study, the recruitment of the study or the conduct of the study. Researchers do not know the identities of the study participants.

### Statistical analysis

Statistical analyses were performed using the SPSS 26.0 software package (IBM, Armonk, NY). Briefly, summary statistics are presented as frequencies and percentages. Categorical data were compared using χ^2^ analysis by Fisher exact test; Continuous data were compared using t test. OS was obtained by the Kaplan–Meier method for different groups. The log-rank test was used to compare survival curves. Variables that showed a significant association with survival were included in multivariate analysis based on the Cox proportional-hazard model. The Chi-square test was applied in the stratified analysis. All significance tests were two tailed, *P* < 0.05 were considered statistically significant.

In the second exploratory analysis for the prognostic value of HR-HPV infection for CSCC patients, propensity score matching (PSM) was used to reduce selection bias from confounding factors between the groups of HR-HPV negative and HR-HPV positive. PSM accounted for age, FIGO stage, SCC-Ag levels, histological grade, Deep stromal invasion (DSI), LVSI, lymph node metastasis and postoperative therapy. Matching was performed in a blinded manner (1:1 ratio, caliper distance = 0.005) without replacement using SPSS 26.0. See procedure for PSM described in Fig. 1.

## Results

### Overall prevalence of HPV infections by age and FIGO stage

There were 1529 patients with FIGO stage I to IIA cervical cancer were identified in the hospital database between 2008 and 2012. Among all these patients, 204 cases were excluded by incomplete medical records, without primary surgery, without HPV test or with non-SCC histology type. A total of 1425 patients with early-stage CSCC who received primary surgery were included in this study for analysis of the prevalent distribution of HPV infections.

**Fig. 2:**
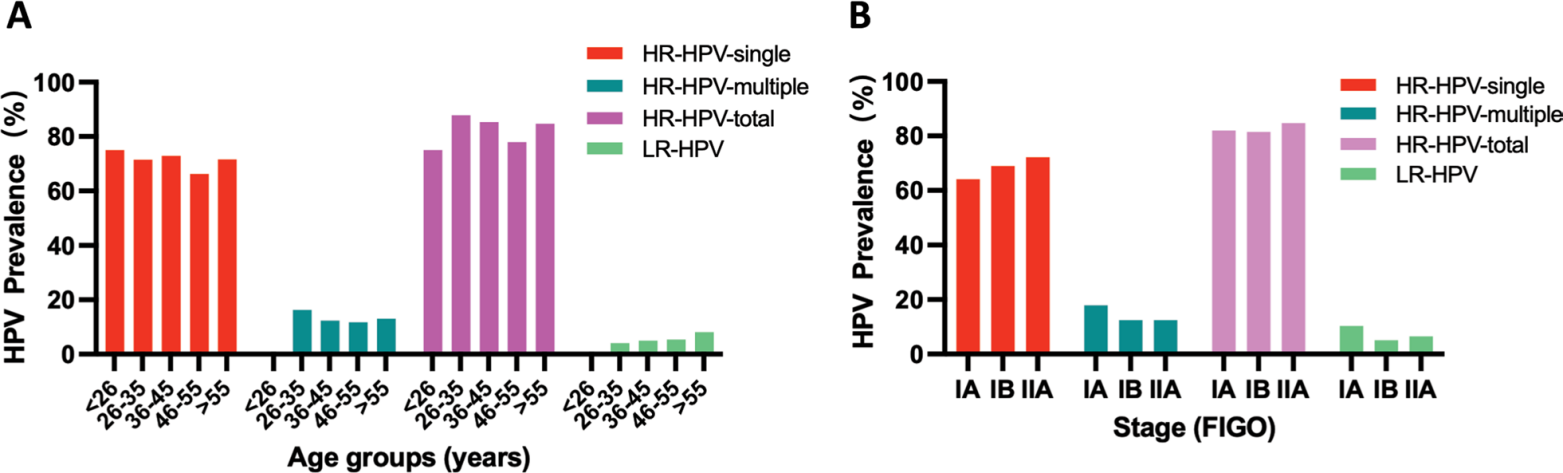
Prevalent distribution of single, multiple, total HR‐HPV and LR-HPV infections in different groups divided by different ranges of age (**A**) and FIGO stages (**B**)

Overall, of the 1425 patients, 1201 (84.3%) cases were positive for HPV infection, including 1178 (82.6%) with carcinogenic HR-HPV genotypes and 82 (5.8%) with LR-HPV genotypes. Among the HR-HPV infections, 998 (70.0%) patients harbored single-type and 180 (12.6%) obtained multiple-type infections (Table 1). When the patients were divided into five groups according to age, the prevalence of total HPV and HR-HPV were generally proportional (Table 1 and Fig. 2A). Similarly, neither total HPV nor HR-HPV did not exhibit significant FIGO stage-specific prevalent distribution (Table 2 and Fig. 2B).

**Table 1:** The age-specific prevalence of HPV infections in early-stage CSCC patients

Age (at surgery)	N	HPV (−), (%)	HPV (+), (%) (LR or HR)	HR-HPV (+), %			LR-HPV ( +)
				Single	Multiple	Subtotal	%
< 26	4	1 (25.0)	3 (75.0)	3 (75.0)	0	3 (75.0)	0
26–35	123	15 (12.2)	108 (87.8)	88 (71.5)	20 (16.3)	108 (87.8)	5 (4.1)
36–45	461	62 (13.4)	399 (86.6)	336 (72.9)	57 (12.4)	393 (85.3)	23 (5.0)
46–55	517	104 (20.1)	413 (79.9)	342 (66.2)	61 (11.8)	403 (78.0)	28 (5.4)
> 55	320	42 (13.1)	278 (86.9)	229 (71.6)	42 (13.1)	271 (84.7)	26 (8.1)
Total cases	1425	224 (15.7)	1201 (84.3)	998 (70.0)	180 (12.6)	1178 (82.6)	82 (5.8)

*HR-HPV* high risk human papillomavirus, *LR-HPV* low risk human papillomavirus

**Table 2:** The FIGO stage-specific prevalence of HPV in early-stage CSCC patients

Stage	N	HPV (−), (%)	HPV (+), (%) (LR or HR)	HR-HPV (+), %			LR-HPV (+), %
(FIGO 2009)				Single	Multiple	Subtotal	
IA	39	6 (15.4)	33 (84.6)	25 (64.1)	7 (17.9)	32 (82.0)	4 (10.3)
IB	864	146 (16.9)	718 (83.1)	596 (69.0)	108 (12.5)	704 (81.5)	44 (5.1)
IIA	522	72 (13.8)	450 (86.2)	377 (72.2)	65 (12.5)	442 (84.7)	34 (6.5)
Total cases	1425	224 (15.7)	1201 (84.3)	998 (70.0)	180 (12.6)	1178 (82.6)	82 (5.8)

*FIGO* International Federation of Gynecology and Obstetrics, *HR-HPV* high risk human papillomavirus, *LR-HPV* low risk human papillomavirus

### Prevalence of HR-HPV subtypes and the single or multiple infection patterns

To explore the prevalent distribution of different HR-HPV genotypes in these early-stage CSCC patients, 13 subtypes of HR-HPV were detected, among which HPV16 was the dominant prevalent genotype with a positive infection status in 929 (65.1%) patients, followed by the other top four subtypes HPV58 (8.7%), 18 (7.7%) and 52 (4.5%) (Table 3). And the infection rates of HPV31, 33, 59, 68, 45, 39, 51, 35 and 56 were relatively rare (Table 3).

**Table 3:** Distribution of HR-HPV sub-genotypes with single and multiple infections

HR-HPV	HR-HPV (+), %			Single/Subtotal
Genotype	Single	Multiple	Subtotal	%
16	773 (54.2)	156 (10.9)	929 (65.1)	83.2
18	70 (4.9)	39 (2.7)	109 (7.7)	64.2
31	22 (1.5)	28 (2.0)	50 (3.5)	44.0
33	18 (1.3)	23 (1.6)	41 (2.9)	43.9
35	0	2 (0.1)	2 (0.1)	0
39	1 (0.1)	8 (0.6)	9 (0.6)	11.1
45	10 (0.7)	4 (0.3)	14 (1.0)	71.4
51	1 (0.1)	4 (0.3)	5 (0.4)	20.0
52	32 (2.2)	32 (2.2)	64 (4.5)	50.0
56	1 (0.1)	1 (0.1)	2 (0.1)	50.0
58	60 (4.2)	64 (4.5)	124 (8.7)	48.4
59	6 (0.4)	9 (0.6)	15 (1.1)	40.0
68	4 (0.3)	12 (0.8)	16 (1.1)	25.0
Total cases	998 (70.0)	180 (12.6)	1178 (82.6)	84.7

*HR-HPV* high risk human papillomavirus

For the infection pattern, of our 1178 cases with positive HR-HPV, the percentage of multiple (co-infection with two or more genotypes) infections was 15.3% in total, which is much less than that of single infections with 84.7%. In the specific subtypes of HPV16, 58, 18, 52 and others, the multiple infections were accounted for 16.8%, 51.6%, 35.8%, 50.0% and were shown in Table 3, respectively.

### Distribution and association of HR-HPV genotypes with various baseline characteristics

The baseline characteristics of our entire cases and the distribution of the top four prevalent HR-HPV genotypes (HPV16, 58, 18 and 52) in groups divided by these clinical features are displayed in Table 4. The association of total HR-HPV (data of each HR-HPV genotypes were not shown) infections with various characteristics were explored by χ^2^ analysiswith Fisher exact test. Age, FIGO stage, histological grade, DSI (less or greater than one half deep stromal invasion), para-aortic/pelvic lymph node metastasis (negative or positive) and postoperative therapy (none or yes) were unrelated to HR-HPV positivity, whereas the presence of HR-HPV positivity exhibited a significant association with SCC-Ag levels (*P* = 0.006, HR = 1.47; 95% CI, 1.12–1.94) and LVSI status (negative or positive) (*P* = 0.003, HR = 1.54; 95% CI, 1.17–2.03) (Table 4).

**Table 4:** Baseline characteristics of the study patients with HPV infections

Characteristic	Mean (SD) or N (%)	HPV16 N (%)	HPV58	HPV18 N (%)	HPV52 N (%)	HR-HPV (13 subtypes)		
			N (%)			N (%)	*P* value	HR (95% CI)
Age (years)								
Mean (SD)	48.1 (9.4)	47.0 (9.4)	51.9 (10.0)	47.6 (8.7)	54.1 (11.4)	48.0 (9.5)	0.257	–
≤ 48	795 (55.8)	569 (71.6)	47 (5.9)	66 (8.3)	21 (2.6)	671 (84.4)	0.057	–
> 48	630 (44.2)	360 (57.1)	77 (12.1)	43 (6.8)	43 (6.8)	507 (80.5)		
FIGO stage								
IA1–IB2	903 (63.4)	590 (65.3)	64 (7.1)	72 (8.0)	39 (4.3)	736 (81.5)	0.146	–
IIA1–IIA2	522 (36.6)	339 (64.9)	60 (11.5)	37 (7.1)	25 (4.8)	442 (84.7)		
SCC-Ag (ng/ml)								
Mean (SD)	4.2 (7.2)	4.3 (7.5)	4.0 (6.3)	4.3 (8.7)	3.7 (7.3)	4.2 (7.3)	0.647	–
< 1.5	608 (42.7)	375 (61.7)	47 (7.7)	54 (8.9)	29 (4.8)	483 (79.4)	0.006	1.47 (1.12–1.94)
≥ 1.5	817 (57.3)	554 (67.8)	77 (9.4)	55 (6.7)	35 (4.3)	695 (85.1)		
Histological grade								
G1	199 (14.0)	134 (67.3)	9 (4.5)	7 (3.5)	7 (3.5)	160 (80.4)	0.364	–
G2–G3	1226 (86.0)	795 (64.8)	115 (9.4)	102 (8.3)	57 (4.6)	1018 (83.0)		
Deep stromal invasion								
Superficial (< 1/2)	586 (41.1)	388 (66.2)	46 (7.8)	50 (8.5)	19 (3.2)	477 (81.4)	0.319	–
Deep (≥ 1/2)	839 (58.9)	541 (64.5)	78 (9.3)	59 (7.0)	45 (5.4)	701 (83.6)		
LVSI								
Negative	681 (47.8)	402 (59.0)	64 (9.4)	61 (9.0)	37 (5.4)	541 (79.4)	0.003	1.54 (1.17–2.03)
Positive	744 (52.2)	527 (70.8)	60 (8.1)	48 (6.5)	27 (3.6)	637 (85.6)		
Para-aortic/Pelvic LN								
Negative	1174 (82.4)	760 (64.7)	106 (9.0)	92 (7.8)	54 (4.6)	969 (82.5)	0.854	–
Positive	251 (17.6)	169 (67.3)	18 (7.2)	17 (6.8)	10 (4.0)	209 (83.3)		
Postoperative therapy								
None	523 (36.7)	332 (63.5)	41 (7.8)	36 (6.9)	24 (4.6)	420 (80.3)	0.081	–
Yes	902 (63.3)	597 (66.2)	83 (9.2)	73 (8.1)	40 (4.4)	758 (84.0)		
Total cases	1425	929 (65.1)	124 (8.7)	107 (7.7)	64 (4.5)	1178 (82.6)		

*FIGO* International Federation of Gynecology and Obstetrics, *HR-HPV* high risk human papillomavirus, *HR* hazard rate, *LN* lymph node, *LVSI* lymph-vascular space invasion, *SCC-Ag* serum squamous cell carcinoma antigen

### Univariate and multivariate analyses of prognostic factors for survival

**Fig. 3:**
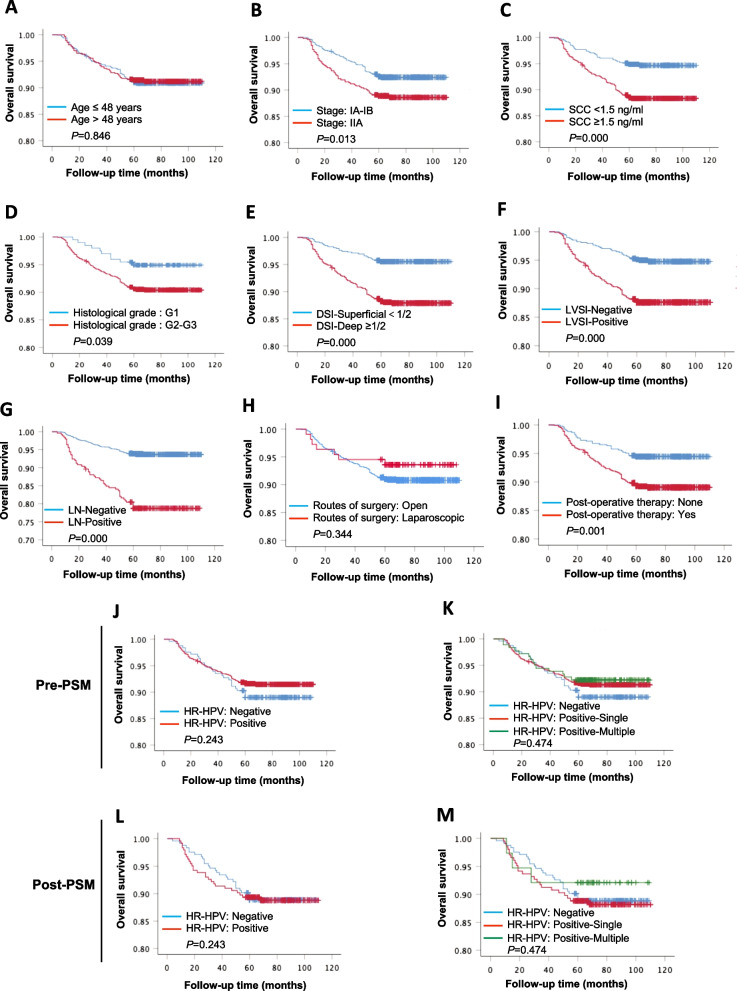
Kaplan–Meier overall survival curves in early-stage cervical cancer patients undergoing primary surgery (n = 1425)according to different groups by various clinicopathological factors, including age (**A**), FIGO stage (**B**), SCC-Ag levels (**C**), histological grade (**D**), DSI (**E**), LVSI (**F**), LN metastasis (**G**), Routes of surgery (**H**), post-operative therapy (**I**), HR-HPV infection status before PSM (**J**), HR-HPV infection patterns before PSM (**K**), HR-HPV infection status after PSM (**L**), HR-HPV infection patterns after PSM (**M**). *DSI* deep stromal invasion, *FIGO* International Federation of Gynecology and Obstetrics, *HR-HPV* high risk human papillomavirus, *LN* lymph node, *LVSI* lymph-vascular space invasion, *PSM* propensity score matching, *SCC-Ag* serum squamous cell carcinoma antigen

The median follow-up time of patients was 76 months (range, 4–110 months).A total of 127 deaths occurred, and the 5-year OS rate was 91.1% (1298/1425). The 5-year OS in different groups of patients divided by various clinicopathology factors were listed in Table 5. Univariate analyses were performed using the log-rank test in each subgroup. FIGO stage, SCC-Ag level, histological grade, DSI, LVSI, lymph node metastasis and post-operative therapy were significantly associated with 5-year OS, whereas age (using a cut off value of 48 years), HR-HPV infection status (positive or negative), Routes of surgery (open or laparoscopic) (*P* = 0.344), HR-HPV infection status (positive or negative) (*P* = 0.243) and patterns (single or multiple) (*P* = 0.474) exhibited no significant relation with survival (Table 5 and Fig. 3A–K). In particular, there were also no significantly 5-year survival association observed in patients with infection of HPV16 (*P* = 0.057), HPV58 (*P* = 0.505), HPV18 (*P* = 0.225) or HPV52 (*P* = 0.768) subtype (Table 5). The potentially survival associated factors were further included to perform multivariate analysis, the results showed that DSI (*P* = 0.007, HR = 1.89; 95% CI, 1.19–2.99), LVSI (*P* = 0.023, HR = 1.63; 95% CI, 1.07–2.48) and lymph node metastasis (*P* = 0.000, HR = 2.56; 95% CI, 1.74–3.75) were independent predictive prognostic factors for 5-year OS in early-stage CSCC patients.

**Table 5:** Univariate and multivariate analysis of potential prognostic factors

Characteristic/Variable	N (%)	5-year OS(%)	Univariate Analysis	Multivariate Analysis	
			*P* value	*P* value	HR (95% CI)
Age (years)			0.846	–	–
≤ 48	795 (55.8)	90.9			
> 48	630 (44.2)	91.3			
FIGO stage			0.013	0.214	–
IA1–IB2	903 (63.4)	92.5			
IIA1–IIA2	522 (36.6)	88.7			
SCC-Ag (ng/ml)			0.000	0.112	-
< 1.5	608 (42.7)	94.7			
≥ 1.5	817 (57.3)	88.4			
Histological grade			0.039	0.576	-
G1	199 (14.0)	95.0			
G2–G3	1226 (86.0)	90.5			
Deep stromal invasion			0.000	0.007	1.89 (1.19–2.99)
Superficial (< 1/2)	586 (41.1)	95.6			
Deep (≥ 1/2)	839 (58.9)	88.0			
LVSI			0.000	0.023	1.63 (1.07–2.48)
Negative	681 (47.8)	94.9			
Positive	744 (52.2)	87.6			
Para-aortic/Pelvic LN			0.000	0.000	2.56 (1.74–3.75)
Negative	1174 (82.4)	93.7			
Positive	251 (17.6)	78.9			
Routes of surgery					
Open	1315 (92.3)	90.9	0.344	–	–
Laparoscopic	110 (7.7)	93.6			
Post-operative therapy			0.001	0.684	–
None	523 (36.7)	94.1			
Yes	902 (63.3)	89.1			
HR-HPV status			0.243	–	–
Negative	247 (17.4)	89.1			
Positive	1178 (82.6)	91.5			
HR-HPV pattern			0.474	–	–
Single	998 (70.0)	91.4			
Multiple	180 (12.6)	92.2			
HPV16 status			0.057	–	–
Negative	429 (30.0)	89.1			
Positive	929 (70.0)	92.1			
HPV58 status			0.505	–	–
Negative	1301 (91.3)	91.2			
Positive	124 (8.7)	89.5			
HPV18 status					
Negative	1316 (92.7)	91.3	0.225	–	–
Positive	109 (7.7)	88.1			
HPV52 status					
Negative	1361 (95.5)	91.0	0.768	–	–
Positive	64 (4.5)	92.2			
Total cases	1425	91.1			

*FIGO* International Federation of Gynecology and Obstetrics, *HR-HPV* high risk human papillomavirus, *LN* lymph node, *LVSI* lymph-vascular space invasion, *OS* overall survival, *SCC-Ag* serum squamous cell carcinoma antigen

Considering the unrelated prognostic significance of HR-HPV infections in our data analysis, and the controversy existed in previous studies, we next performed a PSM to reduce the selection bias for further confirmation. After PSM, a final cohort of 488 patients (244 with each of HR-HPV negative or positive) were eligibly included for further correlation analysis. The patients’ characteristics after PSM were comparable between the two groups with HR-HPV negative or positive (Table 6). The median follow-up time of patients was 78 months (range 4–110 months). The 5-year OS rates in the HR-HPV negative and positive groups were 88.9% and 88.9% respectively (*P* = 0.939). Similarly, no difference observed in HPV positive with single infection and multiple infection groups (88.3% vs 92.1%, *P* = 0.800) (Table 7 and Fig. 3L, M).

**Table 6:** Propensity score-matched baseline characteristics among negative versus positive status of HR-HPV infection

Characteristic	Post-PSM (HR-HPV)		
	(−), N (%)	(+), N (%)	*P* value
Age (years)			
Mean (SD)	48.7 (8.6)	48.3 (9.1)	0.614
≤ 48	122	133	0.365
> 48	122	111	
FIGO stage			
IA1-IB2	164	143	0.061
IIA1–IIA2	80	101	
SCC-Ag (ng/ml)			
Mean (SD)	4.0 (6.7)	3.8 (6.3)	0.775
< 1.5	124	124	1.000
≥ 1.5	120	120	
Histological grade			
G1	37	37	1.000
G2–G3	207	207	
Deep stromal invasion			
Superficial (< 1/2)	107	100	0.583
Deep (≥ 1/2)	137	144	
LVSI			
Negative	138	138	0.585
Positive	106	106	
Para-aortic/Pelvic LN			
Negative	197	401	0.478
Positive	47	87	
Postoperative therapy			
None	102	109	0.584
Yes	142	135	
Total cases	244	244	

*FIGO* International Federation of Gynecology and Obstetrics, *HR-HPV* high risk human papillomavirus, *LN* lymph node, *LVSI* lymph-vascular space invasion, *OS* overall survival, *PSM* propensity score matching, *SCC-Ag* serum squamous cell carcinoma antigen

**Table 7:** Propensity score-matched subgroup analyses for the potential association of HR-HPV status with 5-year overall survival

Groups	N (%)	5-year OS (%)	*P* value
HR-HPV status			
Negative	244 (50.0)	88.9	0.939
Positive	244 (50.0)	88.9	
Single	206 (42.2)	88.3	0.800
Multiple	38 (7.8)	92.1	

*HR-HPV* high risk human papillomavirus, *OS* overall survival

## Discussion

Though it is believed that most cervical cancers were attributed to HR-HPV infections, the prognostic correlation of HPV infections with patient’s survival remains diverse in different data settings from different studies. And the prevalent distribution of HPV infection varies in different geographic regions. In our study, we retrospectively extracted the medical recording data of 1425 patients with early-stage CSCC who underwent radical surgery from Hangzhou province in China, we described the prevalent distribution and subtype patterns of HPV infections, explored the associated clinicopathological parameters with HPV infections, and analyzed the potential predictive biomarkers of 5-year survival factors such as HPV infection and other clinicopathological factors. We found that, (1) the overall infection rate of HPV was 84.3%, including 13 HR-HPV and 8 LR-HPV genotypes with infection rates of 82.6% and 5.8%, respectively; (2) The distribution of HPV infection were proportional in patients across different age and FIGO stages; (3) Pattern of single infections were much more than the multiple infections (70% vs 12.6%), HPV16, 58, 18 and 52 were the dominant top 4 sub-genotypes with infection rates of 65.1%, 8.7%, 7.7% and 4.5%, respectively; (4) SCC-Ag levels and LVSI were statistically associated with HPV status; (5) the 5-year OS of early-stage CSCC patients were related to their clinicopathological factors including FIGO stage, SCC-Ag level, histological grade, DSI, LVSI, lymph node metastasis and post-operative therapy, but not HR-HPV infection status or patterns in both of pre-PSM and post-PSM cohorts. Among them, DSI, LVSI and lymph node metastasis were independent prognostic factors.

Overall, the HPV positivity rate of 84.3% in our population with early-stage cervical cancer is relatively lower than that in previously reported studies with rates of 95.1% [11] or 90.9% [8], it may be, at least partially, due to geographic variation since our data also suggests a significant local variation regarding the prevalent distribution features. Besides, the difference maybe also contributed to different methods of specimen sampling used for HPV genotyping in different studies. Specifically, samples of exfoliated cervical cells collected using a disposable cytology brush were used in our study, whereas, formalin-fixed paraffin-embedded tissue specimens were used in other studies. Therefore, the relatively high rate of non-HPV positivity described in our study and its correlation with patients’ prognosis needs further confirmation by more epidemiological data and experimental studies. For the sub-genotype, HPV16, 58, 18 and 52 were the dominant top 4 sub-genotypes in our selected population with early-stage CSCC, which was basically consistent with the data on the whole population with cervical lesions from our previous study [14], indicating a 9-valent vaccine (Gardasil 9, Merck) would be the suggestion. As previously reported, the pattern of multiple infection was very common in cervical cancers [6, 7], however, our data showed that single infections were much more than the multiple infections. Another important topic at the present study is whether there is an association between HPV status and clinical parameters, of the factors evaluated, χ^2^ analysis revealed SCC-Ag levels and LVSI were found to have a statistically significant relationship with HPV status. More importantly, the prognostic role of HR-HPV infection in SCC is still controversial from a line of previous studies [10, 11, 16–18]. Some reports suggested that patients with HPV-positive ICC had better prognoses than HPV negative ones especially for head and neck squamous carcinomas. The possible mechanism were generalized as: (1) HPV-positive tumors have higher radio- and/or chemosensitivity [19, 20]; (2) HPV-positive tumors exhibited less genetic heterogeneity. Specifically, it was reported that far fewer mutations was found in HPV-positive tumors than that of negative ones especially of those genes including TP53, CDKN2A, PTEN, PIK3CA, FBXW7, HRAS and NOTCH1, which involve in various crucial cellular signaling pathways such as EGFR, PI3K-AKT and mTOR [21, 22]; 3) Patients with HPV-positive infection are usually younger and have more favorable epidemiological, performance status, and other clinical and histopathological features [23]. However, in the setting of cervical cancers, various studies indicated that HPV infection may predict either worse or better clinical outcomes or even have no prognostic value. For example, HPV16 positivity predicted poor prognosis and was associated with pelvic node metastases and LVSI [18], and HPV-18 were at increased risk of death and disease recurrence [11]; However, HPV31 and HPV58 subtypes were found to be associated with better survival outcome [24]. Other reports also demonstrated no association between HPV infection and clinical outcomes [25]. In our series, the results showed that the 5-year survival rates in patients with different HR-HPV infection status, patterns and subtypes were similar even after subjection of PSM to reduce selection bias, indicating that HR-HPV infection status had no prognostic value for 5-year OS of patients with early-stage CSCC. However, due to the fact that information of recurrence and metastasis for these patients in our cohorts were incomplete, we did not analyze the relationship between HPV infection and progression-free survival (PFS) for confirmation. Therefore, further study is still required to establish and determine the prognostic value of HR-HPV infection.

For the strengths of our study: (1) we provide high-quality and personal-level data from a provincial cancer center with uniform treatment guidelines and quality control of pathology in initial surgery from Hangzhou; (2) we found that patients 5-year survival probability were not significantly related to HR-HPV infections; (3) DSI, LVSI and lymph node metastasis were independent prognostic factors of 5-year survival, which was consistent with previous evidences. We also have substantial limitations: (1) due to the nature of this study, there was selection bias in our retrospective setting though we performed PSM to balance the cohorts; (2) due to incomplete dataset for relapse in these patients, we did not analyze the association factors for PFS which may helpful for further determination of the association of HR-HPV infection with survival, since the death rate was relatively low in patients with early-stage cervical cancer and the causes of death interfering 5-year survival contain cancer-specific events and noncancer-specific events; (3) no information on post-relapse therapy available in the dataset, also there were some crucial potentially confounding variables unavailable for performing statistical adjustment.

In conclusion, our present study adds to the growing literature on the prevalent distribution of HPV in early-stage CSCC patients, and HR-HPV infections were not related to 5-year OS, other than FIGO stage, LVSI and lymph node metastasis.

## Data Availability

The datasets used and/or analyzed during the current study available from the corresponding author on reasonable request.

## References

[1] Bray F, Ferlay J, Soerjomataram I, Siegel RL, Torre LA, Jemal A (2018). Global cancer statistics 2018: GLOBOCAN estimates of incidence and mortality worldwide for 36 cancers in 185 countries. CA Cancer J Clin.

[2] Cao W, Chen HD, Yu YW, Li N, Chen WQ (2021). Changing profiles of cancer burden worldwide and in China: a secondary analysis of the global cancer statistics 2020. Chin Med J (Engl).

[3] Watson M, Saraiya M, Benard V, Coughlin SS, Flowers L, Cokkinides V, Schwenn M, Huang Y, Giuliano A (2008). Burden of cervical cancer in the United States, 1998–2003. Cancer.

[4] Hu K, Wang W, Liu X, Meng Q, Zhang F (2018). Comparison of treatment outcomes between squamous cell carcinoma and adenocarcinoma of cervix after definitive radiotherapy or concurrent chemoradiotherapy. Radiat Oncol.

[5] Walboomers JMM, Jacobs MV, Manos MM, Bosch FX, Kummer JA, Shah KV, Snijders PJF, Peto J, Meijer CJLM, Munoz N (1999). Human papillomavirus is a necessary cause of invasive cervical cancer worldwide. J Pathol.

[6] Munoz N, Bosch FX, de Sanjose S, Herrero R, Castellsague X, Shah KV, Snijders PJ, Meijer CJ, International Agency for Research on Cancer Multicenter Cervical Cancer Study G (2003). Epidemiologic classification of human papillomavirus types associated with cervical cancer. N Engl J Med.

[7] de Sanjose S, Quint WG, Alemany L, Geraets DT, Klaustermeier JE, Lloveras B, Tous S, Felix A, Bravo LE, Shin HR (2010). Human papillomavirus genotype attribution in invasive cervical cancer: a retrospective cross-sectional worldwide study. Lancet Oncol.

[8] Li N, Franceschi S, Howell-Jones R, Snijders PJ, Clifford GM (2011). Human papillomavirus type distribution in 30,848 invasive cervical cancers worldwide: variation by geographical region, histological type and year of publication. Int J Cancer.

[9] Bhatla N, Aoki D, Sharma DN, Sankaranarayanan R (2021). Cancer of the cervix uteri: 2021 update. Int J Gynaecol Obstet.

[10] Hang D, Jia M, Ma H, Zhou J, Feng X, Lyu Z, Yin J, Cui H, Yin Y, Jin G (2017). Independent prognostic role of human papillomavirus genotype in cervical cancer. BMC Infect Dis.

[11] Lai CH, Chang CJ, Huang HJ, Hsueh S, Chao A, Yang JE, Lin CT, Huang SL, Hong JH, Chou HH (2007). Role of human papillomavirus genotype in prognosis of early-stage cervical cancer undergoing primary surgery. J Clin Oncol.

[12] Zampronha Rde A, Freitas-Junior R, Murta EF, Michelin MA, Barbaresco AA, Adad SJ, Oliveira AM, Rassi AB, Oton GJ (2013). Human papillomavirus types 16 and 18 and the prognosis of patients with stage I cervical cancer. Clinics (Sao Paulo).

[13] Tao PP, Zheng WP, Wang YG, Bian ML (2012). Sensitive HPV genotyping based on the flow-through hybridization and gene chip. J Biomed Biotechnol.

[14] Xu XX, Zhou JS, Yuan SH, Yu H, Lou HM (2015). Distribution of HPV genotype in invasive cervical carcinoma and cervical intraepithelial neoplasia in Zhejiang Province, Southeast China: Establishing the Baseline for Surveillance. Int J Environ Res Public Health.

[15] Abu-Rustum NR, Yashar CM, Bean S, Bradley K, Campos SM, Chon HS, Chu C, Cohn D, Crispens MA, Damast S (2020). NCCN Guidelines insights: cervical cancer, Version 1.2020. J Natl Compr Canc Netw.

[16] Burger RA, Monk BJ, Kurosaki T, Anton-Culver H, Vasilev SA, Berman ML, Wilczynski SP (1996). Human papillomavirus type 18: association with poor prognosis in early stage cervical cancer. J Natl Cancer Inst.

[17] Im SS, Wilczynski SP, Burger RA, Monk BJ (2003). Early stage cervical cancers containing human papillomavirus type 18 DNA have more nodal metastasis and deeper stromal invasion. Clin Cancer Res.

[18] Pilch H, Gunzel S, Schaffer U, Tanner B, Brockerhoff P, Maeurer M, Hockel M, Hommel G, Knapstein PG (2001). The presence of HPV DNA in cervical cancer: correlation with clinico-pathologic parameters and prognostic significance: 10 years experience at the Department of Obstetrics and Gynecology of the Mainz University. Int J Gynecol Cancer.

[19] Mirghani H, Amen F, Tao Y, Deutsch E, Levy A (2015). Increased radiosensitivity of HPV-positive head and neck cancers: molecular basis and therapeutic perspectives. Cancer Treat Rev.

[20] Ziemann F, Arenz A, Preising S, Wittekindt C, Klussmann JP, Engenhart-Cabillic R, Wittig A (2015). Increased sensitivity of HPV-positive head and neck cancer cell lines to x-irradiation +/- Cisplatin due to decreased expression of E6 and E7 oncoproteins and enhanced apoptosis. Am J Cancer Res.

[21] Agrawal N, Frederick MJ, Pickering CR, Bettegowda C, Chang K, Li RJ, Fakhry C, Xie TX, Zhang J, Wang J (2011). Exome sequencing of head and neck squamous cell carcinoma reveals inactivating mutations in NOTCH1. Science.

[22] Stransky N, Egloff AM, Tward AD, Kostic AD, Cibulskis K, Sivachenko A, Kryukov GV, Lawrence MS, Sougnez C, McKenna A (2011). The mutational landscape of head and neck squamous cell carcinoma. Science.

[23] Pan C, Issaeva N, Yarbrough WG (2018). HPV-driven oropharyngeal cancer: current knowledge of molecular biology and mechanisms of carcinogenesis. Cancers Head Neck.

[24] Huang LW, Chao SL, Hwang JL (2004). Human papillomavirus-31-related types predict better survival in cervical carcinoma. Cancer.

[25] Fule T, Csapo Z, Mathe M, Tatrai P, Laszlo V, Papp Z, Kovalszky I (2006). Prognostic significance of high-risk HPV status in advanced cervical cancers and pelvic lymph nodes. Gynecol Oncol.

